# Validation of a Droplet Digital PCR (ddPCR) Assay to Detect Cyanobacterial 16S rDNA in Human Lung Tissue

**DOI:** 10.3390/toxics11060531

**Published:** 2023-06-14

**Authors:** Rachael E. Barney, Guohong Huang, Torrey L. Gallagher, Maeve Tischbein, John DeWitt, Rachel Martindale, Ethan M. P. LaRochelle, Gregory J. Tsongalis, Elijah W. Stommel

**Affiliations:** 1 Dartmouth-Hitchcock Medical Center, Department of Pathology, Geisel School of Medicine at Dartmouth, Lebanon, NH 03756, USA; 2 Dartmouth-Hitchcock Medical Center, Department of Neurology, Geisel School of Medicine at Dartmouth, Lebanon, NH 03756, USA; maeve.tischbein@dartmouth.edu; 3 Department of Pathology, University of Vermont, Burlington, VT 05405, USA

**Keywords:** ddPCR, cyanobacteria, human tissue, 16S, ALS

## Abstract

Cyanobacteria produce a variety of secondary metabolites, including toxins that may contribute to the development of disease. Previous work was able to detect the presence of a cyanobacterial marker in human nasal and broncoalveolar lavage samples; however, it was not able to determine the quantification of the marker. To further research the relationship between cyanobacteria and human health, we validated a droplet digital polymerase chain reaction (ddPCR) assay to simultaneously detect the cyanobacterial 16S marker and a human housekeeping gene in human lung tissue samples. The ability to detect cyanobacteria in human samples will allow further research into the role cyanobacteria plays in human health and disease.

## 1. Introduction

Cyanobacteria, also known as blue-green algae, are prokaryotes found in nearly all types of water environments [1]. There is some evidence that exposure to cyanobacterial blooms, specifically toxin-producing strains, may contribute to the development of the neurodegenerative disease, amyotrophic lateral sclerosis (ALS) [2]. ALS is an incurable disease that affects motor neurons in the brain and spinal cord, leading to patient paralysis and death within 3–5 years of onset. As with cancer, ALS has been described as a multi-step disease where the interaction among genetic, lifestyle, and environmental factors can contribute to disease development [3]. Clusters of ALS patients have been identified near lakes with known cyanobacterial blooms and poor water quality [4,5,6,7]. Moreover, living near waterbodies can serve as an indicator for cyanotoxin exposure and increases the risk of ALS [8,9,10]. Water-related occupations and activities such as water skiing and boating in contaminated waters also increase the risk of this disease [7,11,12].

Although there is evidence for cyanobacterial illness through multiple routes of exposure (ingestion, dermal contact, aerosolization, etc.), research suggests that the aerosolization of cyanobacteria may be an important route for widespread exposure [13]. Sickness from the aerosolization of non-cyanobacteria (such as *Legionella*), as well as other bacterial toxins, has been previously observed [14]. However, meaningful examination of potential human exposure routes for further health-related research requires reliable and, ideally, quantifiable experimental assays. Previous detection methods described by our group [15] used nested polymerase chain reaction (PCR) to detect 16S rDNA, a marker of cyanobacteria. This work demonstrated the presence of cyanobacteria in the nasal passage and bronchoalveolar lavage fluid of New England subjects. To further investigate cyanobacterial aerosolization as a potential route of exposure, we sought to develop a quantitative assay that will ultimately allow researchers to determine not only the presence, but concentration, of 16S rDNA in human tissue.

Droplet digital PCR (ddPCR) can provide a highly precise, absolute quantification of target nucleic acids within a sample. To achieve absolute quantification, the overall PCR reaction is partitioned into thousands of smaller reactions, resulting in target sequences being either present or absent in each partition [16,17]. Partitioning is performed prior to amplification, resulting in ~20,000 oil droplets containing PCR reactions rather than a single PCR reaction, minimizing effects of inhibitors and competition between targets.

Studies have been conducted using ddPCR to quantify cyanobacteria in environmental samples, primarily in water [18,19,20,21,22,23]. Ai et al. used ddPCR to quantify cyanobacteria and cyanotoxin-producing cyanobacteria in water treatment residuals, using specific primers [18]. Te et al. compared the performance of a multiplex assay that detects two bloom-forming cyanobacterial species, *Microcystis* and *Cylindrospermopsis*, using real-time quantitative PCR and ddPCR [19]. Mejbel et al. compared absolute cyanobacterial 16S rRNA concentration from ddPCR and high-throughput sequencing for lake sediment DNA analyses [20]. However, to date we have not found any studies that detect cyanobacterial 16S rDNA in human tissue.

Here we describe the validation of a ddPCR assay that simultaneously detects cyanobacterial 16S rDNA (16S) and the human housekeeping gene, Ribonuclease P/MRP Subunit P30 (RPP30), in human lung samples. Experiments were conducted to evaluate the linear range, analytical sensitivity, analytical specificity, and precision. The development of this assay may benefit future studies examining the potential impact cyanobacteria has on human health through the assessment of human tissue samples.

## 2. Materials and Methods

### 2.1. Bacterial Controls

Two strains of cyanobacteria were purchased from UTEX Culture Collection of Algae (Austin, TX, USA) as liquid algal cultures. *Microcystis aeruginosa*, LB 2385, is a toxin-producing strain of cyanobacteria. *Synechococcus* sp., LB 2390, is a non-toxin-producing strain of cyanobacteria. The liquid cultures were divided into 2 mL aliquots and DNA was extracted with QIAGEN’s (Germantown, MD, USA) DNeasy Blood & Tissue kit (cat #: 69504), following the manufacturer’s protocol for gram-negative bacteria. DNA was also extracted from two different strains of *Escherichia coli*, a strain mix and a DH5α strain, following the same protocol. Genomic DNA from *Staphylococcus aureus* (ATCC # BAA-1556D-5), and *Pseudomonas aeruginosa* (ATCC # 9027D-5) was purchased from ATCC (American Type Culture Collection). Concentrations were obtained using Invitrogen’s (Waltham, MA, USA) dsDNA High-Sensitivity Assay kit (cat #: Q32851) on the Qubit 3.0 fluorometer.

### 2.2. Human DNA

Human DNA (hDNA) was isolated from frozen autopsy lung tissue samples using the DNeasy Blood & Tissue kit, following the manufacturer’s spin-column protocol for tissue. Concentrations were obtained using the dsDNA High-Sensitivity Assay kit on a Qubit 3.0 fluorometer.

### 2.3. Dilutions

DNA from LB 2385 and LB 2390 was initially diluted to 4 ng/mcL in nuclease-free water. Two-fold serial dilutions were then created for both strains in nuclease-free water and 10 ng of hDNA. Dilutions were made to 61 fg/mcL. Two-fold serial dilution of 2 ng/mcL hDNA was performed to 0.03125 ng/mcL. Five dilutions for each set were run in triplicate.

### 2.4. Primers/Probes

Previously published cyanobacterial 16S rDNA primers designed based on conserved domains for all cyanobacterial species were used [24,25]. Primer sequences were submitted to Bio-Rad’s (Cambridge, MA, USA) PrimePCR custom assay service for the addition of the FAM probe. The quencher was changed to Iowa Black from BHQ1, based on Bio-Rad’s recommendation. To quantify human DNA, RPP30 with HEX probe was chosen due to its relative stability within the genome (Table 1).

### 2.5. ddPCR Set Up

Each ddPCR reaction consisted of the following: 11 mcL of Bio-Rad’s 2× ddPCR Supermix for Probes (no dUTP), 1.1 mcL of 16S assay, 1.1 mcL of RPP30 assay, 2.2 mcL of betaine at 5 mol/L (Sigma-Aldrich, Burlington, MA, USA), 1.1 mcL of the disodium salt of EDTA at 20 mmol/L (Sigma-Aldrich), 0.3 mcL of CviQI (10 IU/mcL) restriction enzyme (NEB, Ipswich, MA), molecular grade nuclease-free water, and DNA.

Oil droplets were generated using the Bio-Rad Automated Droplet Generator. The plate was then transferred to a C1000 Touch™ Deep-Well Thermal Cycler (Bio-Rad) for PCR. The plates were incubated at 95 °C for 5 min, followed by 50 cycles of 94 °C for 30 s and 60 °C for 1 min, then 98 °C for 10 min, and cooled to 4 °C for 1 h. The plates were read on a Bio-Rad QX200 droplet reader using the QuantaSoft v1.7.4 software to assess the number of droplets positive for Cyanobacterial 16S rDNA and/or RPP30.

### 2.6. Data Analysis

Droplet data quality was assessed using three metrics prior to analysis. First, the overall number of events (partitions read) were checked to make sure more than 10,000 occurred. Next, fluorescence amplitudes for both targets were assessed using the 1D view to make sure they were consistent across all wells. Abnormal fluorescence amplitudes can indicate that the reaction was not properly mixed or handled, or that precipitates could have formed. Finally, droplet clusters in the 2D view were assessed to see if a spray pattern of droplets at a 45° angle were seen. 

## 3. Results

### 3.1. ddPCR Optimization

Initial experiments consisted of varied parameters to reduce rain, the droplets that fall between the positive and negative clusters. The first run followed Bio-Rad’s recommendations for the ddPCR Supermix Probes (sans dUTP) super mix as a baseline. No separation was seen between the positive and negative droplets for the 16S assay. A thermal gradient was performed to determine if different annealing temperatures reduced rain; an annealing temperature of 60 °C was kept. Betaine and disodium EDTA were added to the reaction to minimize inhibiting factors [22], resulting in two discernable clusters, but there was significant rain. Next, the thermocycler conditions were adjusted. The initial enzyme activation step was shortened from 10 min to 5 min and the number of cycles was increased from 40 to 50. This created a distinct separation between positive and negative clusters. To see if further optimization was possible, restriction enzyme HaeIII was compared to CviQI and it was determined that CviQI gave cleaner droplet reads than HaeIII. 

Thresholds for RPP30 and 16S were set based on the control wells. For 16S, the channel amplitude threshold was set to 1000 and for RPP30, a threshold of 2000 was set.

### 3.2. Linearity

**Figure 1:**
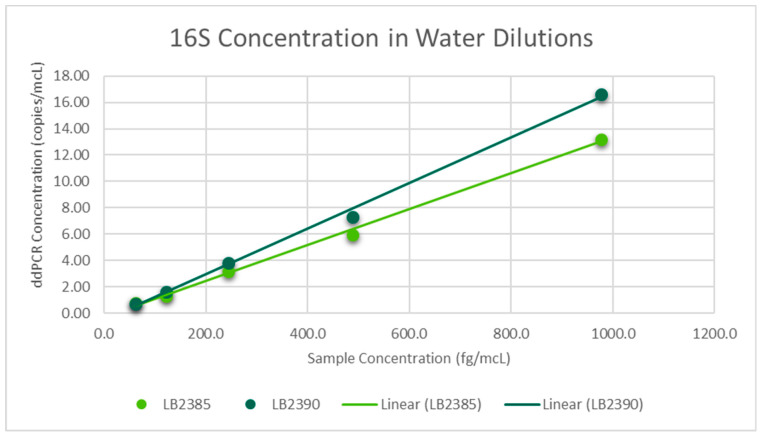
Mean ddPCR 16S concentration (copies/mcL) of LB 2385 and LB 2390 dilutions in water, run in triplicate. LB 2385 R^2^ = 0.9971. LB 2390 R^2^ = 0.9969.

**Figure 2:**
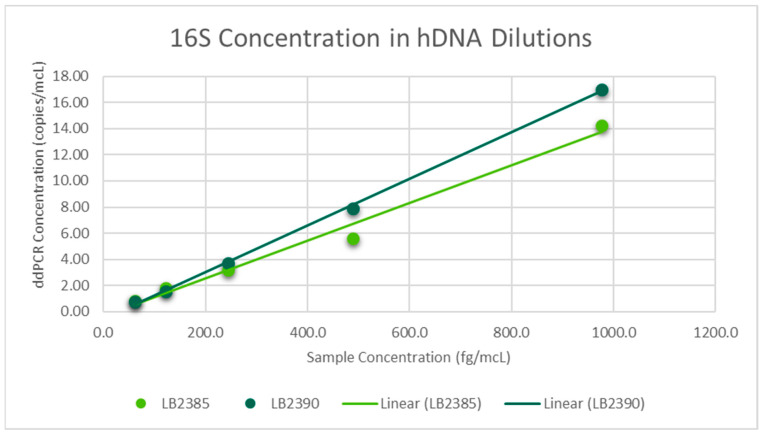
Mean ddPCR 16S concentration (copies/mcL) of LB 2385 and LB 2390 dilutions in human DNA (hDNA), run in triplicate. LB 2385 R^2^ = 0.9853. LB 2390 R^2^ = 0.9991.

**Figure 3:**
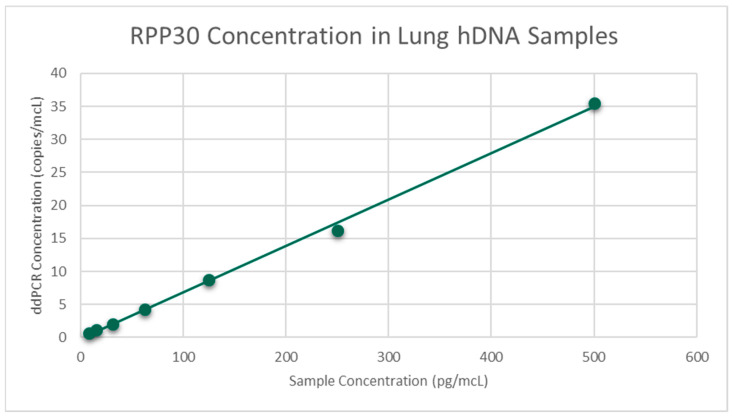
Mean ddPCR RPP30 concentration (copies/mcL) of hDNA dilutions, run in triplicate. R^2^ = 0.9982.

Regression analysis was performed via Excel using the sample concentration and the mean of triplicate ddPCR concentrations. For 16S dilutions in water, linear regression was the best fit for both LB 2385 and LB 2390. A scatter plot was then generated and the R^2^ value was greater than 0.99 for both (Figure 1). Linear regression was also the best fit for LB 2385 and LB 2390 16S dilutions in hDNA. A scatter plot was then generated and the R^2^ value was greater than 0.98 for both (Figure 2). For RPP30, linear regression was the best fit and the generated trend line’s R^2^ value was 0.9982 (Figure 3).

### 3.3. Analytical Sensitivity

**Figure 4:**
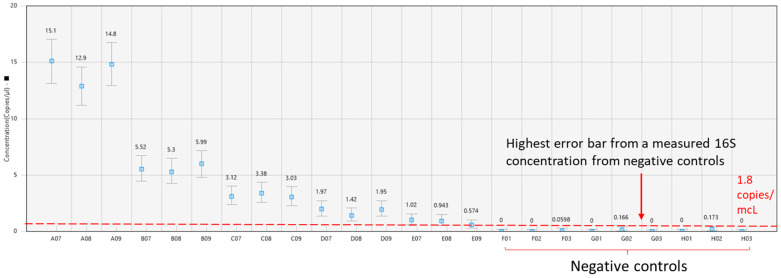
16S limit of detection (LoD) for LB 2385 in hDNA is shown (1.8 copies/mcL). LB 2385 was diluted in 10 ng hDNA, with each dilution run in triplicate. Negative controls included no template controls and *E. coli* DNA tested at 1 pg/mcL.

The limit of detection (LoD) was determined based on the negative template control wells (Figure 4). For LB 2385, the estimated LoD in water is 1.3 copies/mcL, and in hDNA it is 1.8 copies/mcL, both with a total DNA input of 488 fg. For LB 2390, the estimated LoD is 1.6 copies/mcL for both water and hDNA, both with a total DNA input of 488 fg. For RPP30, the estimated LoD is 2–3 copies/mcL, with a total DNA input of 250 pg.

### 3.4. Analytical Specificity

Al-Tebrineh et al. [24,25] previously validated specificity of the 16S rDNA primers both empirically and in silico. To further test the specificity, we also performed ddPCR using strains of *E. coli*, which should not react with the 16S rDNA primer/probes. Fourteen different replicates of *E. coli*, three of which were strain DH5α, were tested and determined to be negative for both targets. The 16S ddPCR concentration mean was 0.04 (a value that is below the experimental LoD), with a standard deviation of 0.06 (which indicates some background noise). The RPP30 ddPCR concentration mean and standard deviation was 0, indicating no significant signal or background noise. Two additional bacterial strains, *Staphylococcus aureus* and *Pseudomonas aeruginosa*, were also negative for both primer/probes (Supplementary Figure S1). Together, these data suggest that the established assay is highly specific for cyanobacteria and human tissue.

### 3.5. Analytical Precision

Dilutions for LB 2385 and LB 2390 were run in triplicate to examine within run precision for 16S (Table 2). In general, as cyanobacterial concentrations approached the LoD (122.1 fg/mcL) an increase in %CV can be observed. Between-run precision was assessed by testing LB 2385 at 976.6 fg/mcL and LB 2390 at 488.3 fg/mcL in every run as positive controls. Both LB2385 and LB 2390 showed comparable 16S results across runs (average 11 copies/mcL). 

Dilutions for hDNA were also run in triplicate to examine run precision (Table 3). A similar trend was observed: the closer to LoD, the greater the %CV.

## 4. Discussion

The ddPCR assay presented here is a sensitive and reproducible method to detect small quantities of cyanobacterial DNA within frozen human lung samples. ddPCR could be potentially used to detect trace amounts of other bacteria within human samples, provided the appropriate primers/probes are developed and validated. While there might be little clinical utility of this particular assay, it does demonstrate the ability to detect low levels of bacteria in human samples. Thus, this ddPCR assay could be used to detect bacterial presence in human blood and tissue samples, such as brain tissue and skin tissue, where toxicological effects from produced toxins might be very important. Further research into the potential impact cyanobacteria has on human health and disease could be explored with this assay.

Previous studies have indicated a link between cyanobacteria exposure and human health conditions, such as ALS. However, the route of exposure, mechanism (e.g., cyanobacterial toxin or other cyanobacteria component) and capacity for negative health impacts (e.g., acute vs. chronic condition) are not well understood. Thus, this assay, paired with additional techniques, may serve to interrogate the role and nature of cyanobacterial impacts on human health and disease.

## 5. Conclusions

We have described the validation of a ddPCR assay that simultaneously detects cyanobacterial 16S rDNA (16S) and the human housekeeping gene, Ribonuclease P/MRP Subunit P30 (RPP30), in human lung samples. Experiments were conducted to evaluate the linear range, analytical sensitivity, analytical specificity, and precision. The development of this assay may benefit future studies examining the potential impact cyanobacteria has on human health through the assessment of human tissue samples. Cyanobacterial neurotoxins have been linked to neurodegeneration and the presence of cyanobacteria in human lung may suggest an important exposure route and how ubiquitous such exposure may be.

## Figures and Tables

**Table 1:** Primer/probe sequences.

Assay ID	Amplicon Location	Oligo ID	Oligonucleotide Sequence (5’ to 3’)	Amplicon Length (bp)	Supplier (Catalog Number)
16S	16S ribosomal RNA	16S Forward	AGCCACACTGGGACTGAGACA	80	Bio-Rad (10031276)
		16S Reverse	TCGCCCATTGCGGAAA		
		Probe	FAM-CCTACGGGAGGCAGCAGTGGG-Iowa Black		
RPP30	hg19|chr10:92660373-92660495:+	ddPCR Copy Number Assay: RPP30, Human, Homo sapiens	Oligonucleotide sequence not provided. HEX reporter; Iowa Black Quencher	67	Bio-Rad (10031243, Assay dHsaCP2500350)

**Table 2:** 16S precision results, displayed as mean (%CV).

Sample	Concentration (fg/mcL)	16S in Water	16S in hDNA
LB 2385	976.6	13.2 (9.2)	14.3 (8.4)
	488.3	5.9 (13.6)	5.6 (6.4)
	244.1	3.2 (9.1)	3.2 (6.6)
	122.1 *	1.3 (41.0)	1.8 (19.2)
LB 2390	976.6	16.6 (8.6)	17.0 (7.1)
	488.3	7.3 (9.6)	7.9 (2.5)
	244.1	3.8 (20.3)	3.7 (8.2)
	122.1 *	1.6 (22.5)	1.6 (29.8)

* = Limit of Detection.

**Table 3:** RPP30 precision results, displayed as mean (%CV).

Sample	Concentration (pg/mcL)	RPP30
Lung hDNA	500	35.5 (3.4)
	250	16.3 (2.3)
	125	8.8 (6.3)
	61.5 *	4.2 (15.7)

* = Limit of detection.

## Data Availability

Not applicable.

## Supplementary Materials

The following supporting information can be downloaded at: https://www.mdpi.com/article/10.3390/toxics11060531/s1, Figure S1 displays additional specificity data.
