# Supplementary material for: Validation of a Droplet Digital PCR (ddPCR) Assay to Detect Cyanobacterial 16S rDNA in Human Lung Tissue
Source: Toxics. 2023 Jun 14;11(6):531. doi: 10.3390/toxics11060531 (PMC10302173; doi:10.3390/toxics11060531)
Supplement: Supplementary file 1 [file toxics-11-00531-s001.zip › SM-Cyano specificity.pptx]

## Slide 1
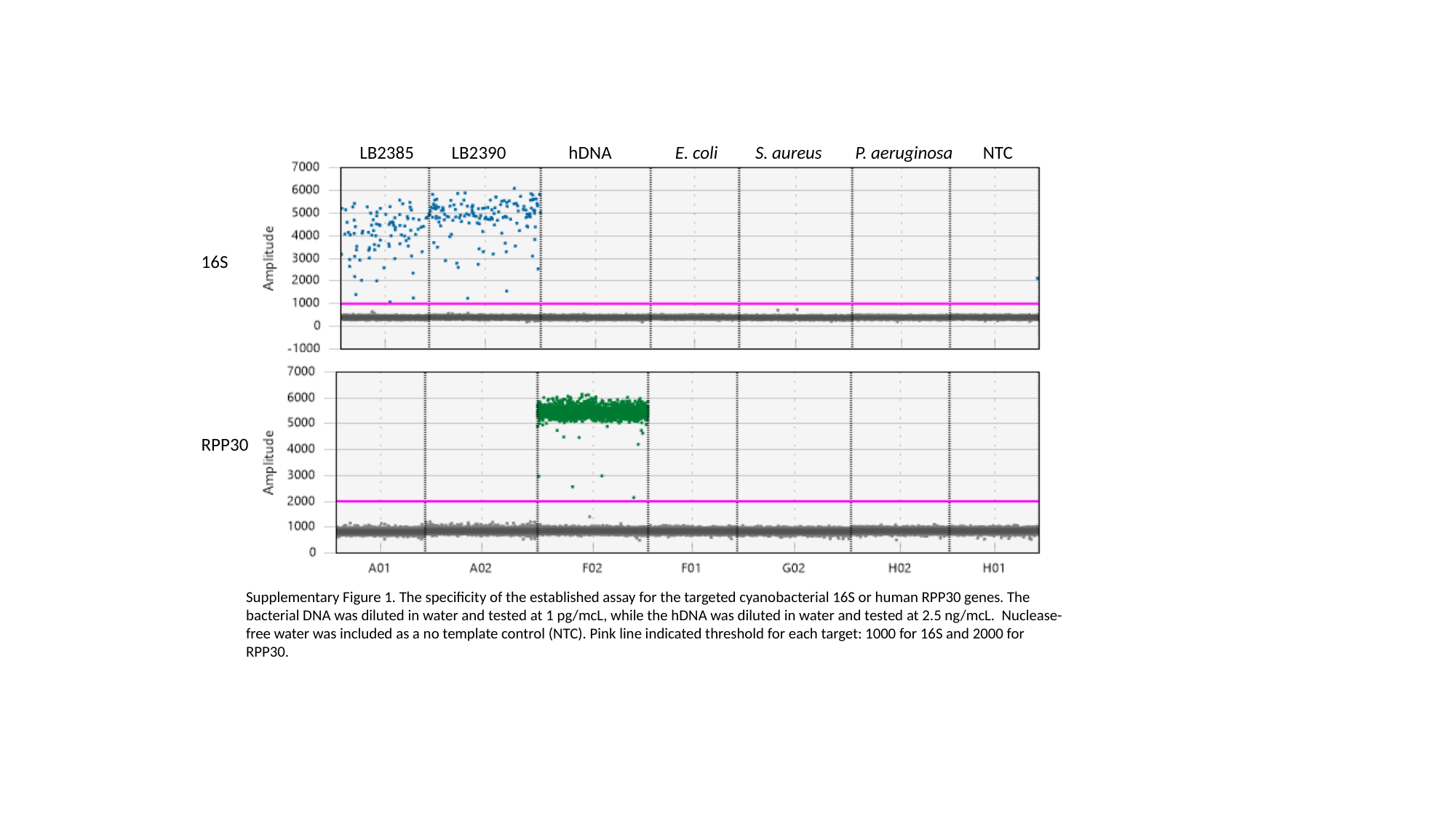

LB2385 LB2390 hDNA E. coli S. aureus P. aeruginosa NTC
16S
RPP30
Supplementary Figure 1. The specificity of the established assay for the targeted cyanobacterial 16S or human RPP30 genes. The bacterial DNA was diluted in water and tested at 1 pg/mcL, while the hDNA was diluted in water and tested at 2.5 ng/mcL. Nuclease-free water was included as a no template control (NTC). Pink line indicated threshold for each target: 1000 for 16S and 2000 for RPP30.
